# Dietary iron and metal-based growth promoters differentially modulate the gut resistome and *Escherichia coli* virulome in weaned pigs

**DOI:** 10.1186/s40104-026-01399-7

**Published:** 2026-05-09

**Authors:** Shya E. Navazesh, Anneliek ter Horst, Weizhang Wen, Yanhong Liu, David Kiang, Zhirong Li, Alice Yu, C. Titus Brown, Peng Ji

**Affiliations:** 1 Departments of Nutrition, University of California, Davis, CA USA; 2 Departments of Population Health and Reproduction, University of California, Davis, CA USA; 3 Departments of Animal Science, University of California, Davis, CA USA; 4 Food and Drug Laboratory Branch, California Department of Public Health, Richmond, CA USA

**Keywords:** Antimicrobial resistance, Dietary iron supplementation, Enterotoxigenic *E. coli*, Growth promoter, Heavy metal resistance, Pharmacological zinc oxide, Postweaning pig

## Abstract

**Background:**

High levels of zinc oxide (ZnO) and copper sulfate are widely used as alternative growth promoters in postweaning pig diet. However, excessive exposure to these metals may drive co-selection for heavy metal (HMR) and antibiotic resistance (AMR). Nursery diets also contain abundant iron to offset the low bioavailability of plant-derived iron, yet how dietary iron influence gut dysbiosis and microbial resistance in postweaning pigs remains unclear. This exploratory study examined the effects of dietary iron and metal-based growth promoters on the fecal resistome of postweaning pigs using shotgun metagenomics and whole-genome sequencing (WGS).

**Methods:**

Fifty weanling pigs were stratified and randomly assigned to five dietary treatments for 24 d. Experimental diets included a control diet (Con) containing 25, 139, and 141 mg/kg of Cu, Fe, and Zn, respectively, a low-iron diet (LFe, 19 mg Fe/kg), a high-iron diet (HFe, 1,219 mg Fe/kg), a high-copper diet (HCu, 257 mg Cu/kg), and a high-zinc diet (HZn, 2,631 mg Zn/kg, including 2,490 mg Zn/kg from ZnO). All pigs were orally administered with F18 enterotoxigenic *Escherichia coli* (ETEC) on d 13–16. Metagenome sequencing were performed on d 24 fecal DNA (*n* = 24) to identify HMR genes (BacMet Predicted database) and AMR genes (CARD database). Functional annotation was performed using HUMAnN3. Whole genome sequencing (WGS) was conducted on 120 *E. coli* isolates from fecal cultures on d 1, 12, and 24, and AMR and virulence genes were identified from contig assemblies using ABRicate.

**Results:**

Dietary metal treatments significantly altered β-diversity of HMR genes compared with Con, with HZn differing from both HCu and LFe (*P* < 0.05). Fecal iron levels correlated with *sodB* (ρ = 0.64, *P* = 0.075), an iron-containing superoxide dismutase*,* while fecal copper levels correlated with *pcoC* (ρ = 0.66, *P* = 0.075), a plasmid-mediated copper resistance gene. Across metagenomes, 172 AMR genes were identified, dominated by glycopeptide and tetracycline resistance. While dietary iron had minimal effects on fecal AMR profile, HZn induced the largest shifts in resistome, including increases of *ant(9)-la*, conferring aminoglycoside resistance on mobile genetic elements, and *adeF*, encoding a multidrug efflux pump (*P* < 0.05). Functional profiling revealed enrichment of carbohydrate metabolism pathways in HZn group (*P* < 0.05). WGS of *E. coli* isolates showed distinct AMR profiles under HZn on d 24 and distinct virulence profile under LFe on d 12, exhibiting increased prevalence of exotoxin and T3SS genes (*P* < 0.05).

**Conclusion:**

Dietary iron restriction enhanced *E. coli* virulence genes, whereas excessive ZnO induced the most pronounced changes in the gut resistome and microbial metabolism, highlighting a risk for AMR co-selection and marked influence on gut microbiota.

**Supplementary Information:**

The online version contains supplementary material available at 10.1186/s40104-026-01399-7.

## Introduction

Antimicrobial resistance (AMR) is a growing public health crisis. In 2021, AMR was directly responsible for an estimated 1.14 million deaths and was associated with nearly 4.71 million deaths globally [1]. The burden continues to escalate, as a recent global surveillance report indicates that AMR rose in over 40% of the pathogen-antibiotic combinations monitored between 2018 and 2023 [2]. If these trends persist, AMR will directly cause 1.91 million deaths and contribute to 8.22 million deaths worldwide by 2050 [1]. Although agriculture is not the sole driver of AMR, recent data suggests that approximately 66% of medically important antimicrobials sold in the U.S. are used in food animal production, primarily swine and cattle [3].

In swine production, early postweaning period is characterized by the highest incidences of enteric infections and diarrheal illness with enterotoxigenic *Escherichia coli* (ETEC) being the leading cause and explaining most therapeutic antibiotic use [4–7]. The ban on using medically important antibiotics for growth promotion in livestock [3, 8, 9] has led to wide adoption of metal-based growth promoters by supplementing pharmacological levels of zinc oxide (ZnO, 2,000–3,000 mg/kg) and, to a less extent, copper sulfate (100–250 mg/kg) to prevent postweaning diarrhea (PWD) [10, 11]. Despite the reported efficacy, the extensive use leads to substantial metal excretion in manure, raising concerns about soil and groundwater accumulation within the farm and broader environment [12–14]. To survive through a heavy metal-rich environment, bacteria have utilized efflux pumps, sequestration and neutralization mechanisms to diminish metal toxicity and enhance tolerance [15]. Some evidence, however, has suggested that the development of heavy metal resistance (HMR) may inadvertently promote AMR through co-selection processes [16]. The proposed mechanisms include 1) co-regulation, where AMR and HMR genes are controlled by the same regulatory network; 2) cross-resistance, where a single gene or mechanism (e.g. efflux pumps) confers resistance to both antibiotics and metals; or 3) co-resistance, where AMR and HMR genes are located on the same mobile genetic element (MGE) and are transferred together. Understanding how alternative growth promoters influence bacterial resistomes in swine production is crucial for controlling PWD while preserving antibiotic efficacy [15].

In contrast to the extensive research on zinc- and copper-based growth promoters, few studies have evaluated the role of iron in modulating gut dysbiosis and microbial resistance in postweaning pigs. As an essential nutrient, the iron “tug-of-war” lies in the center of nutrient competition among host and gut microbiome [17]. Although weaned pigs require 80–100 mg Fe/kg diet, iron concentrations in nursery diets in swine production often exceed this level by 2 to 5 folds [18, 19]. Therefore, intestinal iron flux is likely substantially increased following consumption of postweaning diets. Despite its pro-oxidant properties, dietary supplementation with 1,000 mg Fe/kg did not adversely affect growth performance in young pigs [20]. Furthermore, excessive dietary iron has been shown to alter gut microbial diversity and modulate pathogen virulence [21, 22]. Notably, supranutritional levels of dietary iron attenuated virulence and provided complete protection against even lethal doses of enteric pathogens in a mouse model [21]. In vitro evidence from other pathogens suggests that iron availability can directly affect antibiotic resistance. For example, iron supplementation was shown to reduce metronidazole susceptibility in *Clostridioides difficile* [23]. Similarly, mutations in the iron-responsive transcriptional repressor ferric uptake regulator (*fur*) conferred metronidazole resistance in *H. pylori* [24]*.*

Despite these findings, it remains unclear how changes in dietary iron availability influence AMR profile in postweaning gut microbiota. To address this gap, we investigated the impact of dietary iron and alternative growth promoters on the resistome and microbial functional profiles of postweaning pigs. Because *E. coli* is a key indicator organism for AMR surveillance, we characterized the AMR and virulome profiles of *E. coli* isolates throughout the study.

## Materials and methods

### Study design and sample collection

The animal protocol was reviewed and approved by Institutional Animal Care and Use Committee (protocol#22720) at the University of California, Davis (UC Davis). On weaning day, a total of 50 postweaning piglets (18 gilts/32 barrows, 21–24 days of age) were transferred to the Cole large animal facility at UC Davis. Pigs were stratified by sex and weaning body weight (6.62 ± 1.07 kg) and randomly assigned to one of five dietary treatments (*n* = 10/treatment) for 24 d. Experimental diets included: 1) a control diet (Con) containing 25, 139, and 141 mg/kg of Cu, Fe, and Zn, respectively, 2) a low-iron diet (LFe, 19 mg Fe/kg), 3) a high-iron diet (HFe, 1,219 mg Fe/kg), 4) a high-copper diet (HCu, 257 mg Cu/kg), and 5) a high-zinc diet (HZn, 2,631 mg Zn/kg, including 2,490 mg Zn/kg from ZnO). The LFe, HFe, HCu, and HZn diets had the same ingredient composition as the Con diet, except that the respective minerals were either removed (e.g., LFe) or supplemented at higher levels through adjustments in the vitamin–mineral premix. In the HZn diet, the elevated zinc concentration was achieved by adding ZnO. The Con diet consisted primarily of corn (~ 57%), non-fat dry milk (30%), dried whey (5.4%), and soy protein concentrate (4%). Milk-derived ingredients were included at higher levels than typically used in commercial nursery diets to lower the basal iron concentration. This formulation allowed the LFe diet to be produced by simply removing iron from the premix without altering the primary ingredients. The Con diet met all nutrient requirements for nursery pigs. Ingredient composition and mineral (Fe, Cu, and Zn) concentrations of the experimental diets are presented in Table S1. To prevent iron-deficiency anemia, which may impair growth and immune defense, LFe pigs received intramuscular injections of iron dextran (100 mg Fe/1-mL injection) on d 2 and 7 of the study. Thus, the LFe treatment combined a low-iron diet with parenteral iron supplementation to specifically restrict intestinal iron availability while maintaining systemic iron status, allowing evaluation of its effects on gut resistome profile and colonization resistance to ETEC infection.

Pigs were housed in individual pens (0.61 m × 1.22 m) with free access to feed and water. All pigs were confirmed for genetic susceptibility to F18 ETEC through genotyping *FUT1* polymorphism using the method described in Kruezer et al. [25]. On d 13, all pigs were orally administered with F18 enterotoxigenic *E. coli* (10^10^ CFU/3-mL) once daily for 4 consecutive days (d 13–16). The F18 ETEC was originally isolated from a field disease outbreak by the University of Montreal (isolate number: ECL22131). Fecal samples were collected through rectal swabbing on d 1, 6, 12, 18 and 24 and stored at −80 °C until analysis. This study focused on evaluating dietary effects on fecal resistome, whereas findings regarding clinical signs of ETEC infection, fecal pathogen shedding, growth performance, and gut microbiome will be reported separately.

### Fecal DNA extraction and metagenome sequencing

A subset of 24 fecal samples collected on d 24 were weighed and homogenized. DNA was extracted using the QIAamp PowerFecal Pro DNA Kit (Qiagen, Hilden, Germany) following the manufacturer’s protocol. DNA quality was assessed on a 1% agarose gel electrophoresis with SYBR™ Safe (Thermo Fisher Scientific, Waltham, MA, USA), and concentrations were quantified using a NanoDrop spectrophotometer. Fecal DNA samples were submitted for shotgun metagenomic sequencing at the DNA Technologies and Expression Analysis Core Laboratory at UC Davis using the Illumina NovaSeq 6000 S4 platform. Raw reads were processed using ATLAS (v2.18.1) for quality control and assembly. Quality control was performed using utilities from the BBTools suite and led to an average of 51,681,536 cleaned reads per sample. Cleaned reads were then assembled into contigs using MEGAHIT.

### Fecal resistome profiling

HMR genes were identified from both cleaned reads and assembled contigs using the BacMet-Scan (v.1.0) against the BacMet Predicted database (v.2.0) with Vmatch (v.2.3.1) [26, 27]. The predicted database contains both experimentally validated and predicted HMR genes as well as genes collected using similarity searches in the NCBI non-redundant database. AMR and HMR read counts were rarefied to 274,154 and 6,267 reads, respectively, for alpha- and beta-diversity analysis. Raw counts were used for relative and differential abundance analyses. Genes with relative abundances less than 1% were grouped as “Other”.

AMR genes were also identified from cleaned reads and assembled contigs using the Resistance Gene Identifier (RGI) tool provided by the Comprehensive Antibiotic Resistance Database (CARD) [28]. RGI-*bwt* (v6.0.3) utilized KMA (v1.3.4) to blast reads against the CARD Antibiotic Resistance Ontology Dataset (v3.2.9) and CARD Resistomes and Variants Dataset (v4.0.2) for sequence alignment using the protein homolog model. In contrast, the RGI-*main* (v6.0.3) function first predicts open reading frames (ORFs) from contigs using Prodigal. Identified ORFs were subsequently screened against the CARD database using BLAST, however, the *main* function is also able to predict antimicrobial resistance via point mutations due to its incorporation of the protein variant model. While the RGI analyzes contigs under a Perfect, Strict, and Loose paradigm, only AMR genes categorized as Perfect or Strict matches were retained for analysis.

### Functional annotation

Functional annotation of metagenome data was performed using HUMAnN3 [29] with default settings. HUMAnN3 was used in tandem with the ChocoPhlAn nucleotide database, and UniProt Reference Cluster (UniRef, 2019_01) protein database. The HUMAnN3 pipeline also provided pathway annotation via MetaCyc (v24.0) per sample. Pathway and gene family abundance outputs from HUMANN3 were defaulted to reads per kilobase (RPK) and transformed into copies per million (CPM) to account for any variation in sequencing depth or relative abundance (%) prior to downstream analysis.

### Fecal *E. coli* isolation, whole genome sequencing and identification of AMR and virulence genes

Fecal swabs collected on d 1, 12, and 24 were plated on MacConkey agar for a 24-h culture (37 °C) to grow *E. coli* colonies. *E. coli* colonies were stored in 20% glycerol stock solution at −80 °C, then submitted to the California Department of Public Health for whole genome sequencing. For recovery and confirmation, glycerol stocks were streaked onto TSA-YE and CHROMagar™ EHEC plates. A single colony was then subcultured on TSA-YE and incubated at 37 °C for 18–24 h. Genomic DNA was extracted using the DNeasy Blood and Tissue Kit (Qiagen). Library preparation was performed with the Illumina DNA Prep kit, and sequencing was conducted on the Illumina MiSeq platform using 2 × 251 bp paired-end reads, following CDC PulseNet protocols. FASTQ files were quality-checked using BioNumerics prior to submission to the CDC PulseNet national database. A total of 120 successfully sequenced *E. coli* isolate assemblies were deposited in NCBI Genbank (BioProject: PRJNA277984).

AMR genes and genes encoding virulence factors were screened in contig-level assemblies using ABRicate (v1.0.1) against CARD and the virulence factor database (VFDB). AMR genes were classified according to CARD, whereas virulence genes were categorized according to VFDB criteria [30]*.*

### Statistical analysis

AMR and HMR gene counts were rarefied to the minimum depth using rrarefy in the vegan package (v.2.7-1) in R (v.4.4.3) and analyzed for alpha and beta diversity and relative abundance. A non-metric multidimensional scaling (NMDS) biplot was constructed on Bray–Curtis Dissimilarities between samples. AMR and HMR genes with an adjusted *P*-value of < 0.01 and an absolute Spearman’s correlation value (|ρ|) of > 0.7 and > 0.75 were featured, respectively. A Spearman correlation analysis was also performed between AMR and HMR gene counts and fecal metal concentrations in R. Given the exploratory nature and limited sample size of metagenome analysis, statistical significance was declared at a |ρ| > 0.6 and Benjamini-Hochberg-adjusted *P*-value < 0.10 to identify suggestive associations. Differential abundance of resistance genes counts was analyzed using DESeq2. Pairwise comparisons were extracted and plotted as log_2_ fold-change. Genes with BH-adjusted* P*-values < 0.05 were considered significantly different. Results were visualized using ggplot2 and pheatmap in R.

Relative abundances of functional pathways and gene families predicted from HUMAnN3 were center log-ratio (CLR) transformed using the *compositions* package in R. Principal components analysis (PCA) was performed on the CLR matrix. Group differences were tested on Aitchison distances with PERMANOVA using the *adonis2* package in R, and significance was declared at *P* < 0.05. Differential abundance of functional pathways and gene families were analyzed using MaAsLin2 [31] by applying the LM default settings. Pathways and gene abundances that were present with > 10 CPM in at least one treatment were considered biologically relevant and included in the analysis.

*E. coli* isolates were analyzed using Jaccard distances based on binary presence/absence data of AMR and virulence factor genes using the vegan package in R. A linear mixed effects model was performed on the calculated distances between the post-treatment timepoint to the baseline. Differences within timepoints were additionally tested using PERMANOVA through the *adonis2* and *pairwise.adonis2* package (v.0.4.1) in R. As this is an explorative study with limited sample size, significant association was considered when the BH-corrected *P*-values < 0.10. Descriptive statistics were used to examine the distribution of AMR and virulence factor genes in *E. coli* between treatment groups. To visualize gene presence patterns, heatmaps were generated, showing the percentage of isolates carrying each gene. Heatmaps were produced using the pheatmap package (v.1.0.12), with annotations indicating relevant virulence factor or resistance categories.

## Results

### Metagenome HMR gene profile

**Fig. 1:**
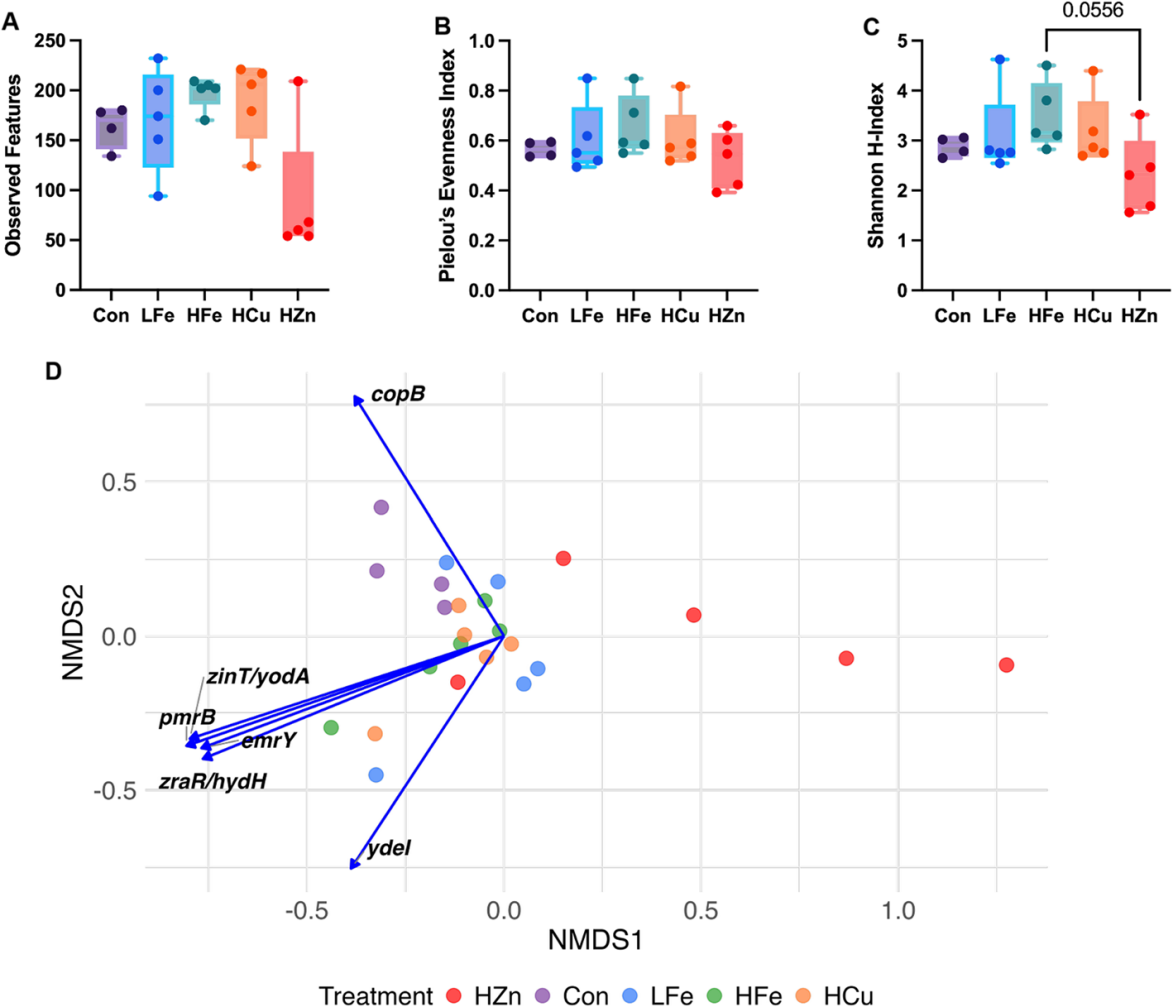
Effects of dietary iron and metal-based growth promoters on fecal heavy metal resistance (HMR) gene diversity. Alpha diversity was assessed using observed features (**A**), Pielou’s evenness (**B**), and the Shannon index (**C**). Beta diversity was evaluated using non-metric multidimensional scaling (NMDS) based on Bray–Curtis dissimilarity (**D**). Spearman correlations between HMR genes and NMDS axes are shown; genes with |ρ| > 0.75 and *P* < 0.01 are displayed as vectors scaled to the correlation coefficient. Con, Control diet; LFe, Low Iron diet; HFe, High Iron diet; HCu, High Copper diet; HZn, High Zinc diet

For metagenome data, contig-based annotation against the BacMet database identified 215 genes associated with metal and/or biocide resistance. At the read level, no differences were observed in the number of observed features or Pielou’s Evenness index. However, the HZn group showed a lower Shannon diversity index compared with HFe group (*P* < 0.05, Fig. 1A–C). Bray–Curtis dissimilarity index revealed that distinct β-diversity among metal-supplemented groups compared with Con group; additionally, HZn differed from HCu and LFe (*P* < 0.05). Correlation analysis between the HMR gene counts and NMDS ordination showed no clear patterns (Fig. 1D).

Differential abundance analysis showed no significant differences in HMR gene patterns among LFe, HFe, and Con (Table S2). HZn treatment produced the largest shifts in HMR gene profiles, and significantly increased *bexA* and reduced *arsC, acn,* and *copB* compared with Con (Fig. S1). In comparison with other metal treatments, HZn had higher abundance of *copY/tcrY*, a transcriptional repressor critical for copper detoxification.

Spearman correlation (|ρ| > 0.06,* P* < 0.10) identified 11 significant correlations between fecal metal concentration and HMR genes (Table 1). Fecal iron was positively associated with *sodB* (ρ = 0.64, *P* = 0.075), an iron-containing superoxide dismutase. Fecal copper positively correlated with *pcoC* (ρ = 0.66, *P* = 0.075), a plasmid mediated copper resistance protein found in *E. coli*. Fecal zinc showed the strongest associations with several genes, including negative correlation with *emhB* (ρ = −0.79, *P* = 0.003), a membrane protein exporter, and *fabV* (ρ = −0.78, *P* = 0.003), an enzyme involved in fatty acid synthesis. Positive correlations were found with *bltD* (ρ = 0.65, *P* = 0.075), a spermidine acetyltransferase, and *emeA* (ρ = 0.64, *P* = 0.075), a multidrug efflux pump.

**Table 1:** Spearman correlation analysis of fecal metal concentrations and heavy metal resistance (HMR) gene abundance in fecal metagenomes

Metal	HMR gene	Correlation coefficient	*P*-value
Fe	*sodB*	0.64	0.075
Cu	*pcoC*	0.66	0.075
Zn	*emhB*	−0.79	0.003
	*fabV*	−0.78	0.003
	*rpoS*	−0.67	0.075
	*copP*	−0.65	0.075
	*mexB*	−0.65	0.075
	*kexD*	−0.63	0.075
	*sdeY*	−0.63	0.075
	*emeA*	0.64	0.075
	*bltD*	0.65	0.075

### Metagenome AMR gene profile

**Fig. 2:**
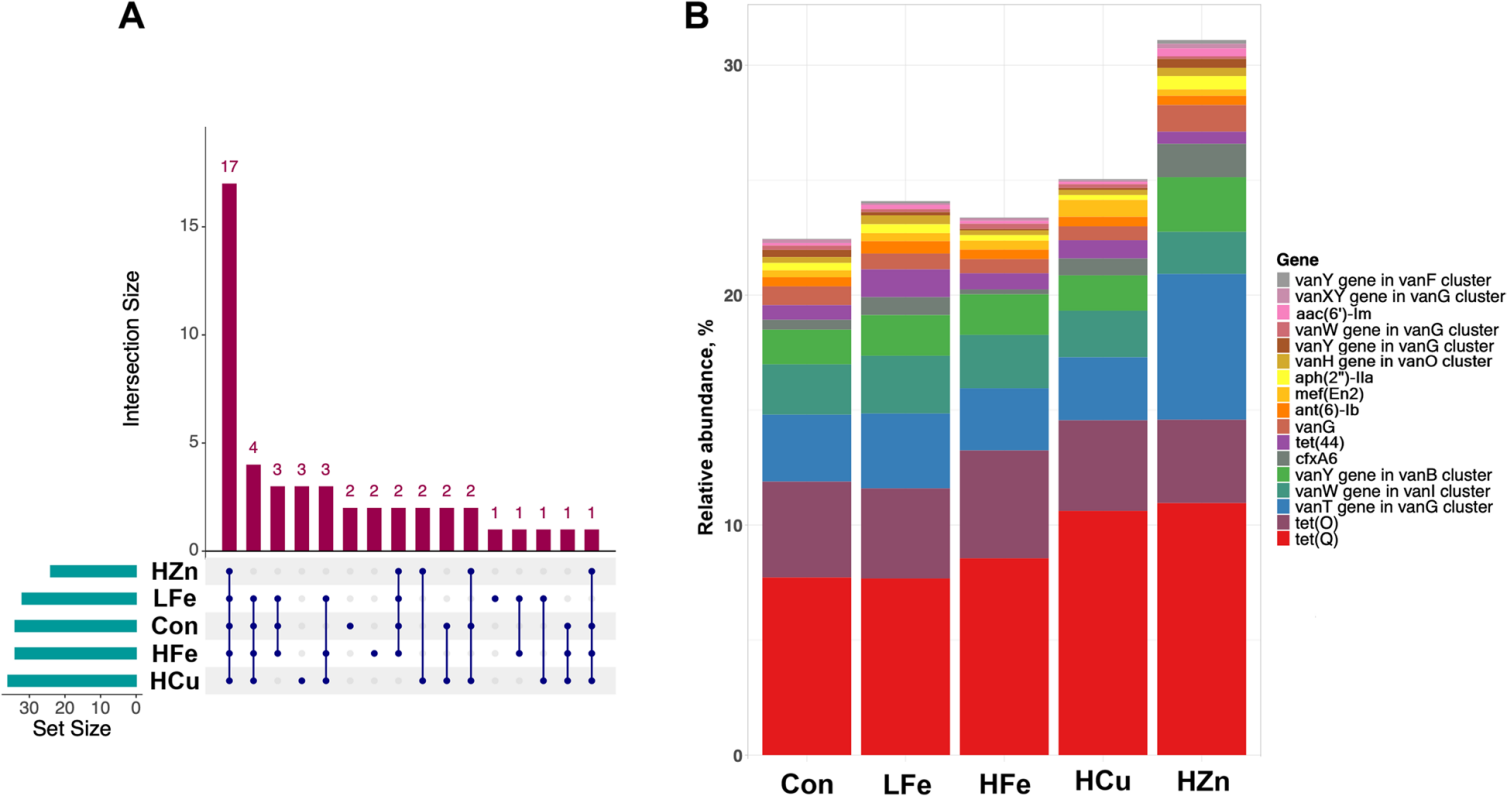
Shared and core antimicrobial resistance (AMR) genes detected in fecal metagenomes across treatments. **A** UpSet plot showing common AMR genes detected within and across treatments, including 17 core genes shared by all samples. **B** Relative abundance of core AMR genes shown as a stacked bar plot. Con, Control diet; LFe, Low Iron Diet; HFe, High Iron diet; HCu, High Copper diet; HZn, High Zinc diet

Contig-based analysis identified 172 unique AMR genes, including 38 classified as multidrug resistant (MDR) genes (Fig. S2, Table S3). Seventeen “core” AMR genes were detected in all samples accounting for approximately 25% of total relative abundance (Fig. 2). Amongst these core genes, *aac(6')-Im*, *ant(6)-Ib*, *tet(44)*, *tet(Q)*, and *tet(O)* are associated with MGE. Abundance of these genes did not differ across treatments.

**Fig. 3:**
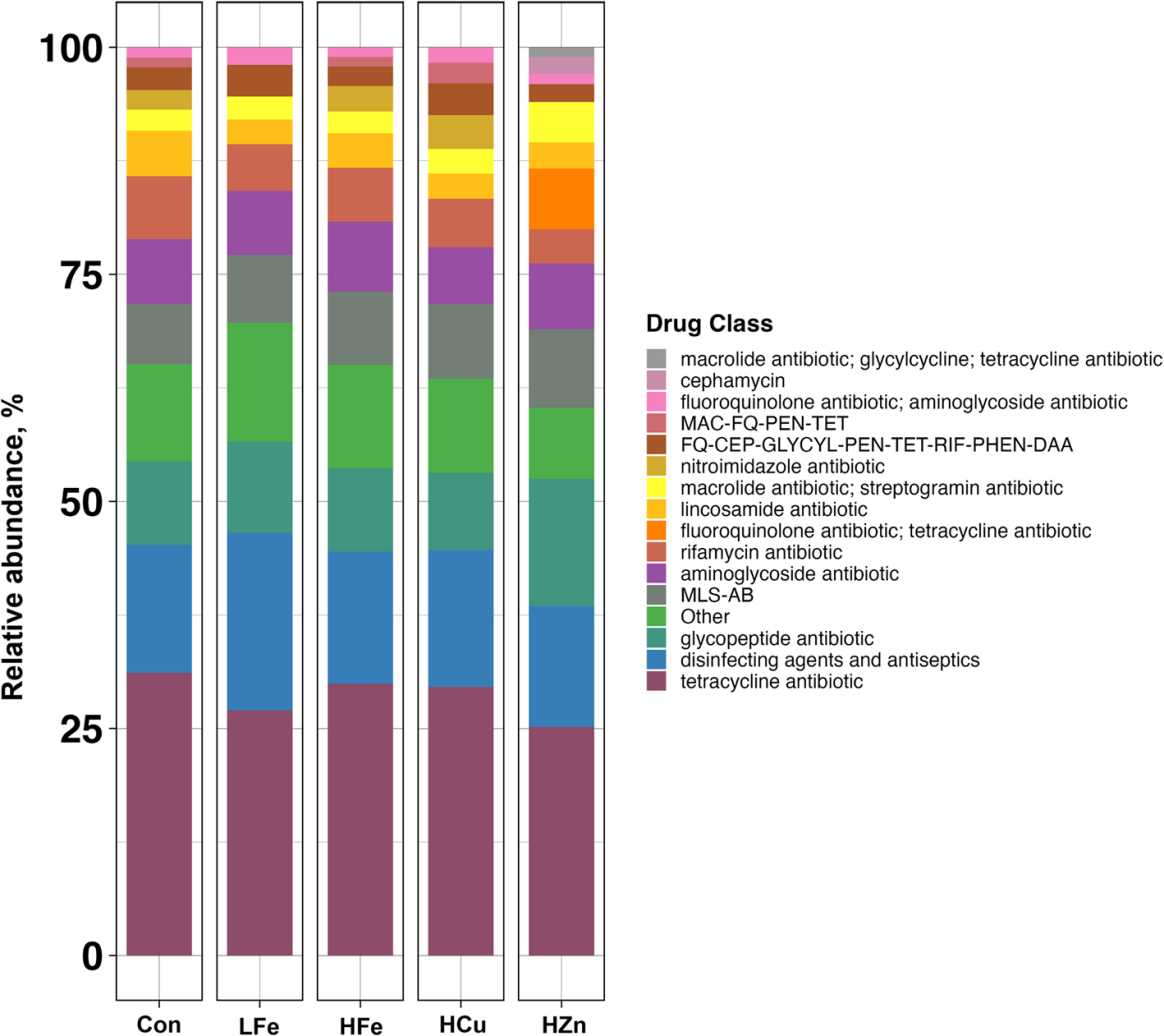
Relative abundance of antimicrobial drug resistance classes in the fecal resistome across treatments. Drug classes representing < 1% of total relative abundance were grouped as “Other”. Abbreviations: MLS-AB, macrolide–lincosamide–streptogramin resistance; MAC-FQ-PEN-TET, macrolide–fluoroquinolone–penam–tetracycline resistance; FQ-CEP-GLYCYL-PEN-TET-RIF-PHEN-DAA, fluoroquinolone–cephalosporin–glycylcycline–penam–tetracycline–rifamycin–phenicol–disinfectant/antiseptic resistance. Con, Control diet; LFe, Low Iron Diet; HFe, High Iron diet; HCu, High Copper diet; HZn, High Zinc diet

**Fig. 4:**
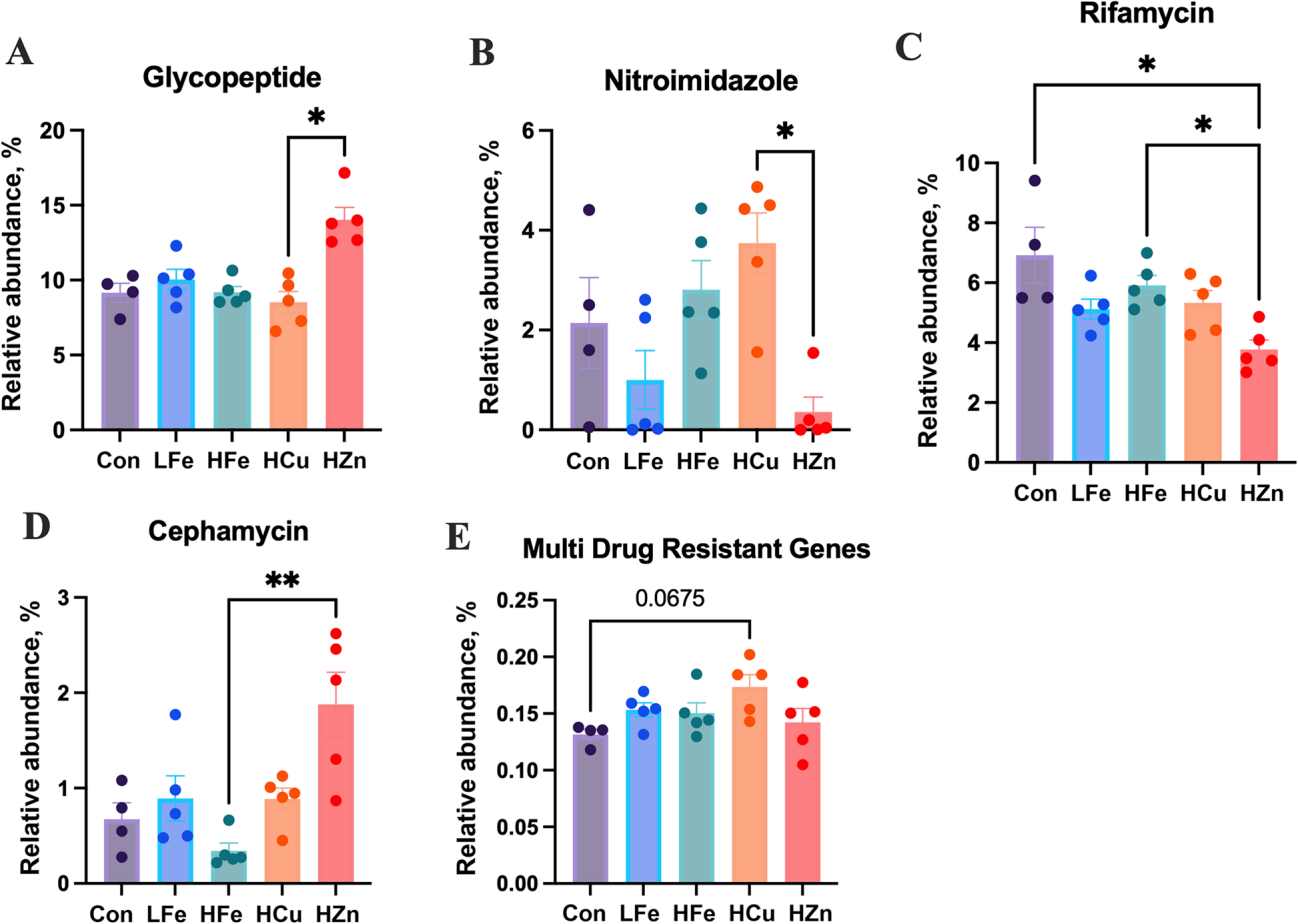
Effects of dietary iron and metal-based growth promoters on the relative abundance of antimicrobial resistance (AMR) genes conferring resistance to glycopeptides (**A**), nitroimidazoles (**B**), rifamycins (**C**), cephamycins (**D**), and multidrug resistance (**E**) in fecal metagenomes. ^*^P < 0.05; ^**^P < 0.01. Con, Control diet; LFe, Low Iron Diet; HFe, High Iron diet; HCu, High Copper diet; HZn, High Zinc diet

Analysis of drug classes at the read level revealed dominance of tetracycline resistance across all groups, followed by resistance to disinfecting agents and antiseptics (DAA), glycopeptides, macrolide-lincosamide-streptogramin-A-streptogramin-B (MLS-AB), and aminoglycoside (Fig. 3). Compared to HCu, HZn had higher abundance of glycopeptide resistant genes and lower nitroimidazole resistant genes (*P* < 0.05, Fig. 4A and B). HZn also had lower abundance of Rifamycin resistant genes compared to Con and HFe, but higher Cephamycin resistant genes relative to HFe (*P* < 0.05, Fig. 4C and D). There was a trend of increased abundance of MDR genes in HCu compared to Con (Fig. 4E, *P* = 0.068).

**Fig. 5:**
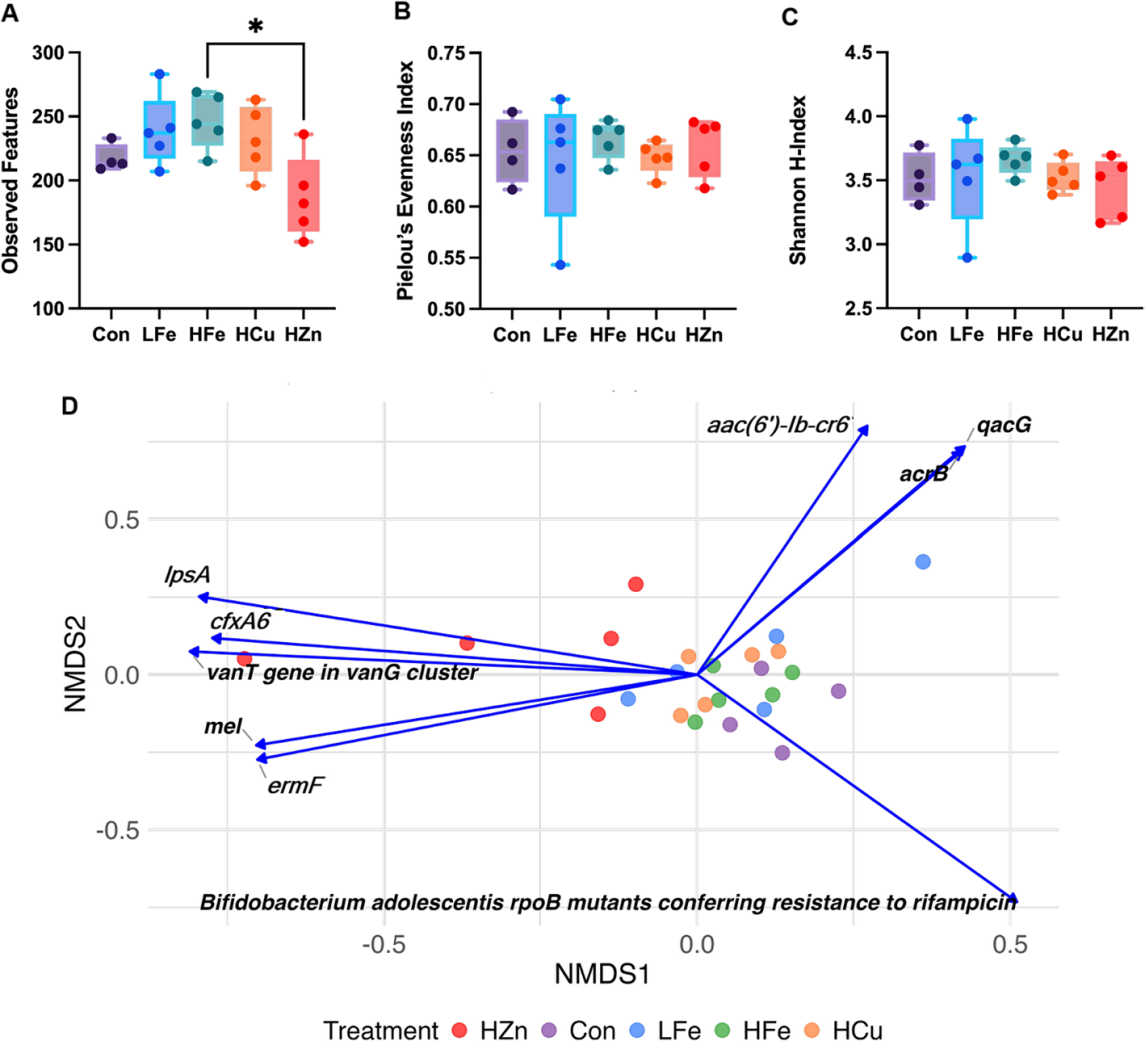
Effects of dietary iron and metal-based growth promoters on fecal antimicrobial resistance (AMR) gene diversity. Alpha diversity was assessed using observed features (**A**), Pielou’s evenness (**B**), and the Shannon index (**C**). Beta diversity was evaluated using NMDS based on Bray–Curtis dissimilarity (**D**). Genes significantly correlated with NMDS axes (|ρ| > 0.75, *P* < 0.01) are shown as vectors scaled to correlation coefficients. ^*^P < 0.05. Con, Control diet; LFe, Low Iron Diet; HFe, High Iron diet; HCu, High Copper diet; HZn, High Zinc diet

Iron availability did not alter α- or β-diversity of AMR genes. However, HFe showed more observed features compared to HZn (Fig. 5A–C). Bray–Curtis dissimilarity revealed a significant treatment effect of HZn (*P* < 0.05), with vector fitting showing *lpsA*, *cfxA6*, *mel*, and the *vanT* (*vanG *gene cluster) as main drivers of group separation (ρ > −0.7, *P* < 0.01, Fig. 5D). Differential abundance analysis revealed no significant changes in AMR genes in pigs fed the HFe or HCu diets, however, *cblA-1*, a *Bacteroides uniformis* specific beta-lactamase, was significantly increased in the LFe and HZn treatment groups relative to the control (*P* < 0.05, Table S4). The HZn group also was shown to have higher abundances *tet(36)* and *ant(9)-Ia* compared to the Con and higher abundances of *adeF***,** compared to all treatment groups (*P* < 0.05). The abundances of *cfxA6*, the *vanT* gene from the *vanG* cluster, and *lnuA*, a plasmid-mediated nucleotidyltransferase, were also significantly higher in the HZn group compared to the HFe group (*P* < 0.05). No correlations were observed between AMR genes and fecal metal concentrations for iron, copper, and zinc.

### Metagenome functional annotation

PERMANOVA of Aitchison distances based on gene families relative abundances revealed differences between HZn and all other groups (*P* ≤ 0.06), between LFe and HFe (*P* = 0.06), and between HCu and Con (*P* = 0.06) and HFe (*P* = 0.06, Fig. S3B). However, overall pathway profiles did not differ across groups (Fig. S3A).

Differential abundance analysis of reads revealed that the LFe and HFe treatment groups did not differ at the pathway level relative to the Con group. The HCu treatment, however, led to significant reductions in glycogen degradation I pathway (GLYCOCAT-PWY, *P* < 0.05, Fig. S3C) and sucrose degradation IV pathway (PWY-5384, *P* < 0.05, Fig. S3D, Table S5) in *Bifidobacterium* relative to the Con group, further confirmed by decrease in the reaction catalyzed by sucrose phosphorylase (SUCROSE-PHOSPHORYLASE-RXN).

The HZn group showed the most changes with 12 altered metabolic pathways relative to the Con group (*P* < 0.05, Table S5). These changes included the enrichment of carbohydrate metabolism, evidenced by increases in the interconnected pathways of GDP-mannose biosynthesis (PWY-5659), starch degradation (PWY-6731), myo-, chiro- and scyllo-inositol degradation pathway (PWY-7237), and galactose and glucuronate metabolism (GALACT-GLUCUROCAT-PWY) (*P* < 0.05, Fig. S4A). Conversely, the gluconeogenesis pathway (PWY-66–399) was significantly decreased in HZn (*P* < 0.05). HZn also impacted energy metabolism (*P* < 0.05, Fig. S4B), including reductions in anaerobic energy metabolism (PWY-7383) and reductive TCA cycle I (P23-PWY) indicating diminished anaerobic capacity in the gut microbiota of the HZn group.

Furthermore, HZn was associated with a decrease in vitamin biosynthesis (*P* < 0.05, Fig. S4C), including folate transformations II pathway (PWY-3841), biosynthesis of 1,4-dihydroxy-2-naphthoic acid (DHNA, PWY-7371) and menaquinol-8 (PWY-7992), the latter two of which are intermediate and final product of vitamin K_2_. Gene family analysis revealed decreased capability for the conversion of 5,10-methylenetetrahydrofolate to 5,10-methenyltetrahydrofolate (RXN 1.5.1.5), and ultimately leading to N10-formyltetrahydrofolate (RXN 3.5.4.9), a crucial molecule for purine biosynthesis (*P* < 0.05, Table S6). HZn also was shown to modulate pathways involved in bacterial metabolism, evidenced by reduction in reads mapped to the ADP-L-glycero-β-D-manno-heptose biosynthesis pathway (PWY0-1241, *P* < 0.05) and the tRNA charging pathway (TRNA-CHARGING-PWY) in *E. coli*.

### AMR and virulence gene profiles of *E. coli* genomes

The number of virulence or AMR genes did not differ between treatments at any timepoints (Fig. S5). To evaluate whether pigs differed in the magnitude of change post-treatment, each sample’s Jaccard distance was calculated to its own baseline (d 1), then a linear mixed effects model analysis was performed. For both virulence factor and AMR gene sets, there was a significant day effect, however, no treatment or interaction effects were observed, indicating that changes occurred over time, but the average magnitude of change was comparable across treatments.

**Fig. 6:**
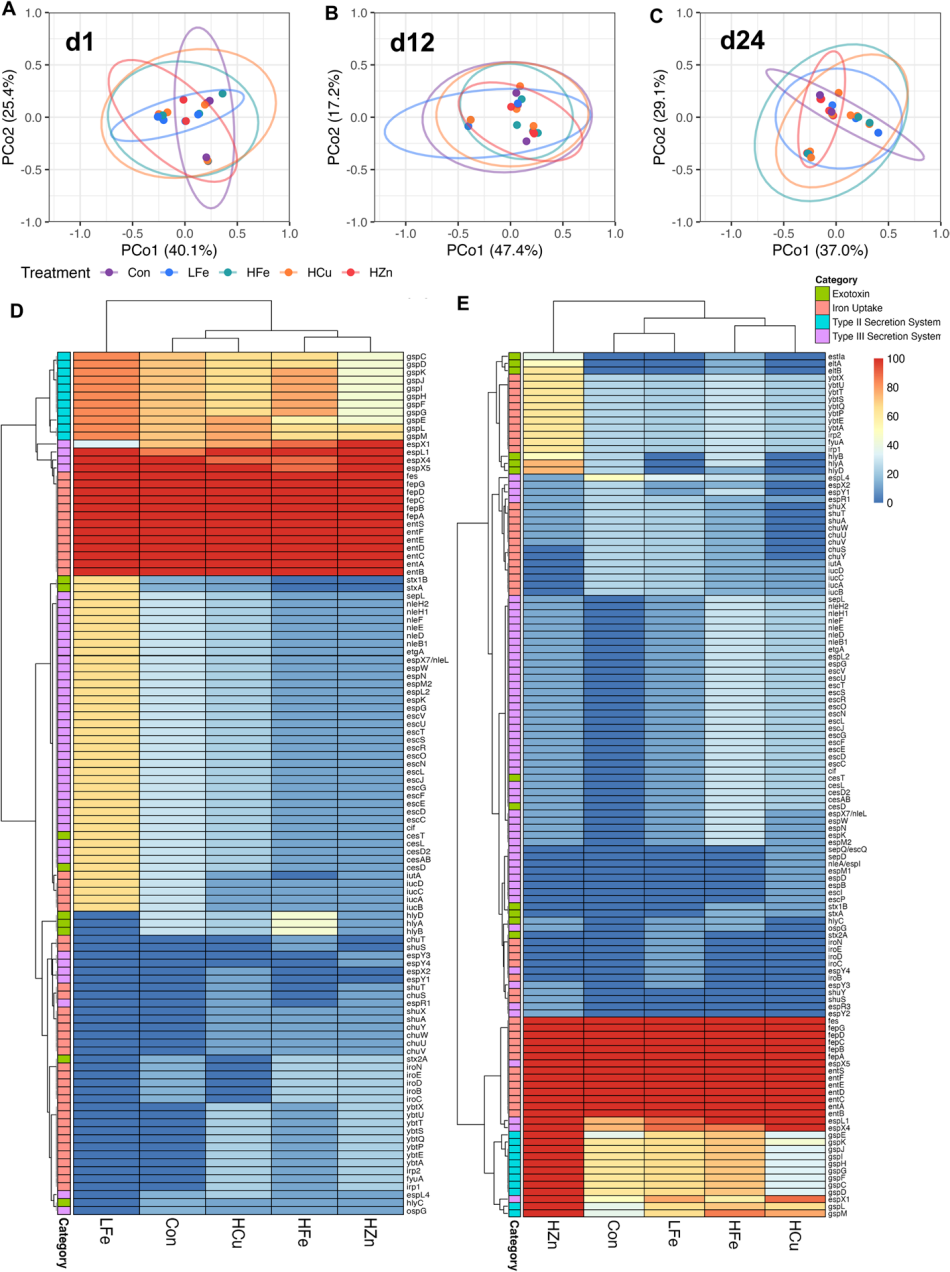
Virulence factor gene profiles in fecal *E. coli* genomes. Principal coordinates analysis (PCoA) of virulence factor gene profiles on d 1 (**A**), 12 (**B**), and 24 (**C**), showing the first two axes with 95% confidence ellipses by treatment. Heatmaps display the prevalence (%) of virulence genes on d 12 (**D**) and d 24 (**E**). Categories significantly different among treatments are shown, with treatments ordered by hierarchical clustering. Con, Control diet; LFe, Low Iron diet; HFe, High Iron diet; HCu, High Copper diet; HZn, High Zinc diet

To further evaluate whether treatments differed in their profiles at individual timepoints, a pairwise PERMANOVA analysis was performed on data subset by day. On d 1, the virulence profile of the Con group differed from the LF, HFe, and HZn treatment groups, indicating variation prior to dietary intervention (*P* < 0.05, Fig. 6A, Table S7). On d 12, the virulence profile of LFe differed from HZn (*P* = 0.05, Fig. 6B) and trended versus HFe (*P* = 0.058) and HCu (0.099). To determine the key drivers of these differences, genes were grouped by virulence categories and analyzed (Table S8). Iron uptake gene profiles of LFe differed (significantly or trended) from HCu (*P* < 0.05), HFe (*P* = 0.06), and HZn (*P* = 0.07, Fig. 6D). Isolates from LFe tended to have higher prevalences of iron uptake cluster (*iuc*) and *iutA* genes in the aerobactin operon. Genes encoding for the type III secretion system (T3SS) and exotoxins were also found to be more prevalent in LFe compared to HFe and HZn (*P* < 0.05). There was a trend for higher prevalence of Shiga toxin genes (*stx*) and lower hemolysin (*hly*) genes in LFe compared to HFe.

On d 24, the virulence profile of HZn differed from that of HCu (*P* < 0.05) and trended towards difference compared to the Con (*P* = 0.064) and LFe (*P* = 0.093, Fig. 6C). HZn *E. coli* genomes had altered exotoxin gene profiles compared to HCu (*P* < 0.05), LFe (*P* < 0.05), and HFe (*P* = 0.08, Fig. 6E). These differences may be attributed to an increased prevalence of hemolysin (*hly*), heat-labile enterotoxin (*elt*), and heat-stable toxin (*estIIa*). HCu isolates showed differences in iron uptake genes (*P* = 0.08) relative to HZn and genes in effector delivery systems relative to HZn (*P* < 0.05) and Con (*P* = 0.09). Further evaluation revealed that the HCu group had altered Type II secretion system (T2SS) genes profiles relative to Con (*P* < 0.05), HZn (*P* < 0.05), and LFe (*P* = 0.07).

**Fig. 7:**
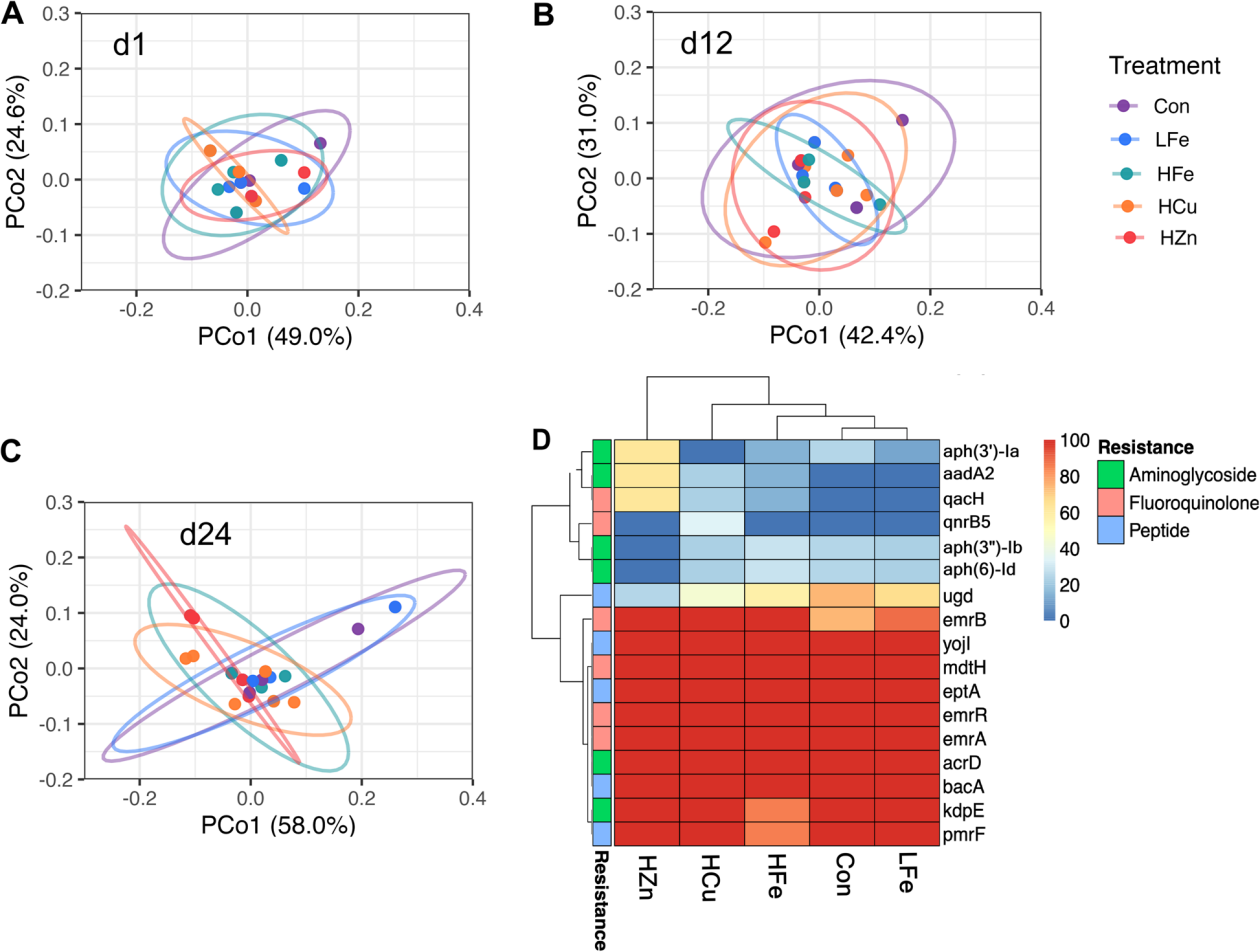
Antimicrobial resistance (AMR) gene profiles in fecal *E. coli* genomes. Principal coordinates analysis (PCoA) of AMR gene profiles on d 1 (**A**), 12 (**B**), and 24 (**C**). PCoA plots show the first two axes with 95% confidence ellipses by treatment. Heatmap shows the prevalence (%) of AMR genes in *E. coli* genomes isolated on d 24 (**D**). Resistance categories significantly different among treatments are displayed, with treatments ordered by hierarchical clustering. Con, Control diet; LFe, Low Iron diet; HFe, High Iron diet; HCu, High Copper diet; HZn, High Zinc diet

Differences in AMR profiles were only observed on d 24, where isolates from the HZn diet significantly differed from Con, LFe, and HCu (*P* < 0.05, Fig. 7A–C) and trended towards significance relative to HFe (*P* = 0.07, Table S9). Upon evaluation of drug classes, the differences were primarily driven by aminoglycoside and fluoroquinolone resistance genes (*P* < 0.05, Table S10). The aminoglycoside profile of the HZn group significantly differed from the LFe and HCu treatment groups (*P* < 0.05) and trended towards significant relative to the Con and HFe groups (*P* < 0.07). Differences in aminoglycoside resistance genes were likely driven by shifts in MGE encoded genes, including increases in *aadA2* gene and *aph(3')-Ia*, and decreases in *aph(3'')-Ib* and *aph(6)-Id* in HZn isolates (Fig. 7D). The fluoroquinolone resistance gene profiles of HZn significantly differed from LFe and Con (*P* < 0.05). Similarly, fluoroquinolone resistance in the HCu group was significantly different compared to the Con group (*P* < 0.05) and approached significance when compared to the LFe group (*P* = 0.053).

## Discussion

### Resistome and functional profile of fecal metagenome

Building on our previous work, we explored how dietary iron and alternative growth promoters influence the fecal resistome in postweaning pigs. The high levels of ZnO and copper substantially increased intestinal metal flux. Like antibiotics, excess metal exposure exerts selective pressures on bacteria, yet its effects on gut microbiome and AMR dissemination remain poorly understood [32]. Pharmacological levels of ZnO (2,100–2,500 mg/kg) have been associated with increased prevalence of AMR genes and multidrug resistant *E. coli* in postweaning pigs [33–37]. Similarly, high copper (200 mg/kg) in nursery diet enriches *E. coli* and increases chloramphenicol and ciprofloxacin resistance [38]. High dietary copper (125–175 mg/kg) was also found to increase abundance of *tcrB* (copper resistance gene) alongside AMR genes for macrolide, tetracycline, and glycopeptides in fecal *Enterococcus* isolates, indicating co-resistance [39, 40].

In our study, metagenomic analysis revealed significant associations, especially in the HZn treatment, between fecal metal concentrations and HMR genes. *sodB* encodes superoxide dismutase B, which detoxifies superoxide radicals and prevents ferrous iron regeneration in Fenton reaction. The mRNA expression of *sodB* is regulated by Fur [41]. The positive association between fecal iron and *sodB* abundance possibly reflects microbial adaptation to increased oxidative stress induced by excessive intestinal iron. Fecal copper positively correlated with *pcoC*, which is a component of the plasmid-borne *pco* operon that mediates copper resistance and was first identified in *E. coli* from pigs fed copper-supplemented diets [16, 42, 43]. Transfer of *pco*-carrying plasmid to *Salmonella* was shown to enhance its resistance to copper toxicity, underscoring the horizontal dissemination within microbial community [44]. Given the fact that plasmids often carry AMR genes, horizontal transmission could facilitate co-selecting metal tolerance and AMR [45]. Interestingly, HZn was negatively associated with multiple HMR genes, although no canonical zinc-resistant genes were detected. It is plausible that these negative correlations reflect the reduced microbial diversity observed in HZn-treated pigs (reported elsewhere), which may have limited the overall abundance of HMR gene carriers.

Contrary to our hypothesis, no significant associations were identified between fecal metal concentrations and AMR genes. This finding differs from environmental studies demonstrating that heavy metal contaminations can promote co-selection of AMR [15]. For example, application of pig manure to agricultural land has been shown to result in copper and zinc accumulation accompanied by increased abundance of β-lactam resistance genes [46] and multi-drug resistance genes (*mdtB* and *yegN*) [47]. The mechanisms underlying the discrepancy between gut and soil microbiota responses to excessive metal exposure remain elusive. However, several factors may contribute. First, exposure duration differs substantially: the gastrointestinal tract represents a relatively short-term and dynamic exposure condition, whereas metal accumulation in soil may exert prolonged and persistent selective pressure on soil microbes. Second, ecological context differ markedly between intestinal and environmental microbiota. Lastly, baseline AMR backgrounds in the study animals, as well as farm-specific or environmental stressors, may influence co-selective pressures.

Across treatments, tetracycline, glycopeptide, macrolide-lincosamide-streptogramin A and B (MLS-AB), and aminoglycoside resistant genes were most prevalent. While dominance of tetracycline resistance agrees with other reports [48, 49], high prevalence of glycopeptide resistance was a novel finding. The lower abundances of β-lactam resistance genes compared to other studies may arise from differences in biosecurity, antimicrobial use, and farm management [50, 51]. At the contig-level, 17 core AMR genes were found in all samples. Vancomycin resistance genes were most abundant. Vancomycin is a glycopeptide antibiotic classified as critically important for human medicine. Its use in food-producing animals has been banned by FDA since 1997 [52]. Since these vancomycin resistant genes were chromosomally associated, the observed pattern may reflect intrinsic resistance retained within microbial community rather than horizontal transfer. Four core AMR genes (*aac(6')-Im*, *ant(6)-Ib, tet(O),* and *tet(Q)*) were associated with MGEs, highlighting risks of horizontal transfer*.* High abundance of *tet(O)* and *tet(Q)* (3.6%–11%) suggests that they have been stably integrated into the porcine gut resistome. At the read level, LFe, HFe and HCu treatments had no effects on diversity or abundance of AMR genes, consistent with the study by Brinck et al. [53], in which high copper supplementation (250 mg/kg) had subtle effects on microbial composition and AMR profiles. In contrast, β-diversity of HZn AMR genes exhibited a distinct cluster, suggesting a significant shift in AMR profile. Although differential abundance analysis detected few changes, the *adeF* and *ant(9)-Ia* genes, which encode a membrane fusion protein of a multidrug efflux pump (AdeFGH) and an aminoglycoside nucleotidyltransferase, respectively, were increased in HZn pigs. AdeFGH was reported to be located in chromosome of *Acinetobacter baumannii*, and the presence of this gene contributes to tetracycline and fluoroquinolone resistance [54]. These drug classes are categorized by the World Health Organization (WHO) as “highly important antibiotics” and “highest priority critically important antibiotics”, respectively, and are authorized for treatments in both humans and animals [55]. Increased abundance of *adeF* therefore may indicate selection for bacteria with greater drug efflux capacity resulting from Zn overexposure. In contrast, *ant(9)-Ia* is frequently detected in MGEs of *Staphylococcus aureus*, *Mammaliicoccus sciuri*, and *Enterococcus faecalis*. In a recent mobilome analysis, the *ant(9)-Ia*/*Tn554*/*erm(A)* association emerged as the most frequent MGE linked multi-AMR gene group in both human and animal-derived *S. aureus* genomes [56]. The similarity in these MGE associated AMR gene configurations suggests dissemination of MGE across various host reservoirs.

Changes in AMR abundance may reflect both selective pressure and changes in microbial composition. Previously, we observed increased fecal *Bacteroides* abundance in HZn pigs, a genus known to harbor AMR and HMR genes, including *bexA* identified in this study. Thus, shifts in resistome likely mirror compositional changes of microbial community, as reported by others [50, 53].

Functional annotation revealed minimal alternations in metabolic pathways in response to altering dietary iron or high copper. This agrees with studies showing limited effects of iron supplementation on microbial metabolism [57, 58]. In contrast, iron restriction is associated with dysbiosis and reduced short-chain fatty acid production in rats and in vitro fermentation of human microbiota [58, 59]. In *E. coli*, iron restriction impairs TCA cycle and respiratory chain reactions, whereas excess iron enhances these pathways [60]. Copper’s oxidative porential and antimicrobial properties appeared to minimally affect metabolic pathways, aside from reduced glycogen and sucrose degradation. Since sucrose degradation was identified with genus *Bifidobacterium*, reduction in this pathway was consistent with decreased *Bifidobacterium* abundance in HCu [61]. In contrast to our findings, others have reported broader impact of copper supplementation (200 mg/kg) on microbial metabolism (e.g., protein, amino acid, and carbohydrate metabolism) in postweaning pigs [38].

HZn, however, broadly altered microbial metabolism, enriching complex carbohydrate degradation pathways (e.g., starch, inositol, and glucuronides). This aligns with transcriptomic evidence showing increased gene expression involving in carbohydrate metabolism in *E. coli* exposed to ZnO nanoparticles [62]. This change is likely driven by increased *Bacteroidota* abundance in the current study [61]. HZn also reduced biosynthesis pathways for DHNA, a key intermediate for bacterial vitamin K_2_ synthesis, and menaquinol-8, which is a reduced form of vitamin K_2_ (menaquinone). Menaquinone is a lipid-soluble electron carrier that is essential to electron transport chain and ATP production in all Gram-positive bacteria and anaerobic respiration of Gram-negative bacteria [63]. Together with the decrease in reductive TCA cycle, these changes indicated that HZn significantly impairs bacterial energy metabolism, growth and proliferation, which may mechanistically explain the growth-promoting and infection-preventing effects of ZnO. Furthermore, HZn decreased ADP-L-glycero-β-D-manno-heptose biosynthesis. The byproduct of this pathway, ADP-heptose, is an essential component for lipopolysaccharide (LPS) inner core formation in Gram-negative bacteria [64]. Disrupted LPS synthesis can reduce virulence and increase antibiotic susceptibility [65, 66]. HZn also reduced an *E. coli* tRNA charging pathway, implying diminished protein synthesis capacity. Collectively, these findings suggest that HZn imposes antibiotic-like selective pressure that limits growth potential of gut microbes.

### Resistome and virulome of fecal *E. coli* isolates

Iron lies at the center of host-microbe nutrient competition. Bacteria evolve various mechanisms for iron acquisition under restricted conditions. Regulators such as Fur and diptheria toxin repressor (DtxR) not only control iron uptake but also modulate virulence gene expression in response to altered iron availability [67]. Iron starvation has been shown to affect production of colonization factors CFA/I and CS6 in ETEC in vitro [68, 69]. While transcriptional regulation alone cannot fully explain the altered *E. coli* virulence profile observed in LFe and HZn on d 12 and 24, it likely contributes to the expansion of virulent *E. coli* under iron restriction or excessive zinc exposure.

Notably, iron uptake cluster (*iuc*) genes and *iutA* (aerobactin receptor) were more prevalent in LFe isolates, which also exhibited a distinct profile of type III secretion system (T3SS) genes compared with HFe and HZn isolates. T3SS mediates effector proteins delivery into host cells and contributes to the pathogenesis of many Gram-negative pathogens including *Yersinia*, *Salmonella*, *Shigella*, and enteropathogenic and enterohemorrhagic *E. coli*. [70, 71]. In LFe isolates, T3SS genes of the *esc* cluster, *esp*, *nle*, and *ces* were more prevalent. These genes have been observed both within and outside of the locus of enterocyte effacement, indicating intact T3SS machinery and enhanced host interaction potential. Iron-dependent activation of Fur represses Shiga-like toxins (*sltA* and *sltB*) and hemolysin genes in *E. coli* [72, 73]. In the current study, Shiga toxin genes were enriched in LFe isolates, while HFe isolates had less Shiga toxin gene but more hemolysin-related genes. Although these effects were transient, observed primarily on d 12, they coincide with the window of highest risk of PWD [7].

Although effects of excessive copper exposure on *E. coli* virulence is not well understood, studies have linked copper resistance with increased bacterial virulence [74]. For instance, the siderophore yersiniabactin enhances copper resistance and virulence in uropathogenic *E. coli* [75], while loss of copper exporter *ctpV* reduces virulence of *Mycobacterium tuberculosis* [76]. In our study, *E. coli* from HCu pigs on d 24 exhibited reduced type II secretion system (T2SS) gene, which mediates heat-labile enterotoxin secretion in human-infecting ETEC [77]. Conversely, HZn isolates showed higher prevalence of T2SS and exotoxin related genes including hemolysin (*hly*), heat-labile enterotoxin (*elt*), and heat-stable toxin (*estIIa*). Although prior studies reported that ZnO attenuate bacterial virulence [78–80], our findings suggest that ZnO may instead select for more virulent *E. coli* strains or enhance horizontal transfer of virulence genes.

The AMR profiles of HZn isolates diverged at d 24, primarily driven by aminoglycoside- and fluoroquinolone-resistance genes. For example, *aadA2*, an aminoglycoside nucleotidyltransferase gene encoded by plasmids and integrons, is widely distributed in Gram-negative bacteria including *Klebsiella pneumoniae*, *E. coli*, *Citrobacter freundii*, *Salmonella *spp., and *Aeromonas *spp. Similarly, *aph(3')-Ia*, a transposon encoded aminoglycoside phosphotransferase, is commonly identified in *E. coli* and *Salmonella enterica*. Given the ability of these MGEs to disseminate resistance across environments and the prevalence of their host bacteria in both livestock and humans, this finding raises concern for cross-species transmission [81, 82]. Similar trends were observed in *E. coli* isolates from zinc-supplemented (80 mg/d) milk-fed calves, exhibiting altered sensitivity to quinolones [83]. Prolonged ZnO exposure has also been linked to resistance against aminoglycosides, cephalosporins, and sulfonamides in *E. coli* isolates [84]. Because aminoglycoside resistance genes were associated with MGEs, co-selection under excessive zinc exposure is plausible. Given the zoonotic potential of *E. coli*, these findings underscore the risk of ZnO-mediated AMR dissemination.

## Conclusion

This study evaluated the effects of dietary iron and metal-based growth promoters on the porcine gut resistome and *E. coli* virulence using metagenomics and WGS. While iron levels had little impact on AMR abundance, iron restriction increased *E. coli* virulence genes, suggesting higher infection risk. High copper correlated with *pcoC* but did not alter AMR profiles. In contrast, high ZnO exposure caused marked shifts in AMR and metabolic functions, supporting a role in AMR co-selection and microbiome modulation.

Study limitations include small sample size and single time-point metagenomic analysis. In our study, metabolic functions of gut bacteria were predicted based on metagenomic data. Future longitudinal and multi-omics studies are needed to clarify how dietary metals influence microbial adaptation, AMR transmission, and ETEC pathogenesis during postweaning.

## Supplementary Information

Additional file 1: Table S1. Ingredient composition and mineral concentrations of experimental diets. Table S2. Differential abundance of heavy metal resistance genes in metagenomes. Table S3. Characteristics of antimicrobial resistance genes identified in fecal metagenome. Table S4. Differential abundance analysis of antimicrobial resistance genes in metagenomes. Table S5. Functional pathway differences relative to control identified by MaAsLin2 using HUMAnN3. Table S6. Differences in functional gene families relative to control identified by MaAsLin2 using HUMAnN3. Table S7. Differences in virulence factor gene profiles of *E. coli* genomes revealed by PERMANOVA analysis. Table S8. Differences in functional categories of virulence genes in *E. coli* genomes revealed by PERMANOVA Analysis. Table S9. Differences in antimicrobial resistance gene profiles in *E. coli* genomes revealed by PERMANOVA analysis. Table S10. Differences in functional categories of antimicrobial resistance genes in *E. coli* genomes revealed by PERMANOVA analysis.

Additional file 2: Fig. S1. Differentially abundant heavy metal resistance (HMR) genes in the HZn group relative to Con (A), LFe (B), HFe (C), and HCu (D). Fig. S2. Distribution of antimicrobial drug classes in the fecal resistome across all metagenomes. Fig. S3. Principal component analysis (PCA) of centered log-ratio–transformed relative abundances of gene families (A) and functional pathways (B). Fig. S4. Functional pathways significantly altered by the HZn diet relative to Con. Fig. S5. Number of virulence genes (A–C) and AMR genes (D–F) detected in isolated E. coli genomes on d 1, 12, and 24.

## Data Availability

The metagenomic sequencing data have been deposited in the NCBI Sequence Read Archive (SRA) database under BioProject ID PRJNA1391520. Project information is accessible in the following link: (https://www.ncbi.nlm.nih.gov/sra/PRJNA1391520). The whole genome sequencing data of cultured **E. coli** isolate assemblies have been deposited in the NCBI GenBank under BioProject accession PRJNA277984.

## Abbreviations

AMR: Antimicrobial resistance
CARD: Comprehensive Antibiotic Resistance Database
CEP: Cephalosporin
CLR: Center log-ratio
CPM: Copies per million
DAA: Disinfecting agents and antiseptics
DHNA: 1,4-Dihydroxy-2-naphthoic acid
ETEC: Enterotoxigenic *Escherichia coli*
FQ: Fluoroquinolone
Fur: Ferric uptake regulator
GLYCYL: Glycylcycline
HMR: Heavy metal resistance
HUMAnN3: Human microbiome project unified metabolic analysis network, version 3
LPS: Lipopolysaccharide
MAC: Macrolide
MDR: Multidrug resistance
MGE: Mobile genetic element
MLS-AB: Macrolide-lincosamide-streptogramin A and B
NMDS: Non-metric multidimensional scaling
ORF: Open reading frame
PCA: Principal components analysis
PEN: Penam
PHEN: Phenicol
PWD: Postweaning diarrhea
RGI: Resistance gene identifier
RIF: Rifamycin
RPK: Reads per kilobase
SOD: Superoxide dismutase
T2SS: Type II secretion system
T3SS: Type III secretion system
TCA: Tricarboxylic acid cycle
TET: Tetracycline
VFDB: Virulence factor database
WGS: Whole genome sequencing
WHO: World health organization
