# Supplementary material for: Dietary iron and metal-based growth promoters differentially modulate the gut resistome and Escherichia coli virulome in weaned pigs
Source: J Anim Sci Biotechnol. 2026 May 9;17:87. doi: 10.1186/s40104-026-01399-7 (PMC13156864; doi:10.1186/s40104-026-01399-7)
Supplement: Supplementary file 2 — Additional file 2: Fig. S1. Differentially abundant heavy metal resistance (HMR) genes in the HZn group relative to Con (A), LFe (B), HFe (C), and HCu (D). Fig. S2. Distribution of antimicrobial drug classes in the fecal resistome across all metagenomes. Fig. S3. Principal component analysis (PCA) of centered log-ratio–transformed relative abundances of gene families (A) and functional pathways (B). Fig. S4. Functional pathways significantly altered by the HZn diet relative to Con. Fig. S5. Number of virulence genes (A–C) and AMR genes (D–F) detected in isolated E. coli genomes on d 1, 12, and 24. [file 40104_2026_1399_MOESM2_ESM.pdf]

Dietary Iron and Metal Growth Promoters Differentially Modulate the Gut Resistome and E. coli Virulome in Weaned Pigs
Supplemental Figures

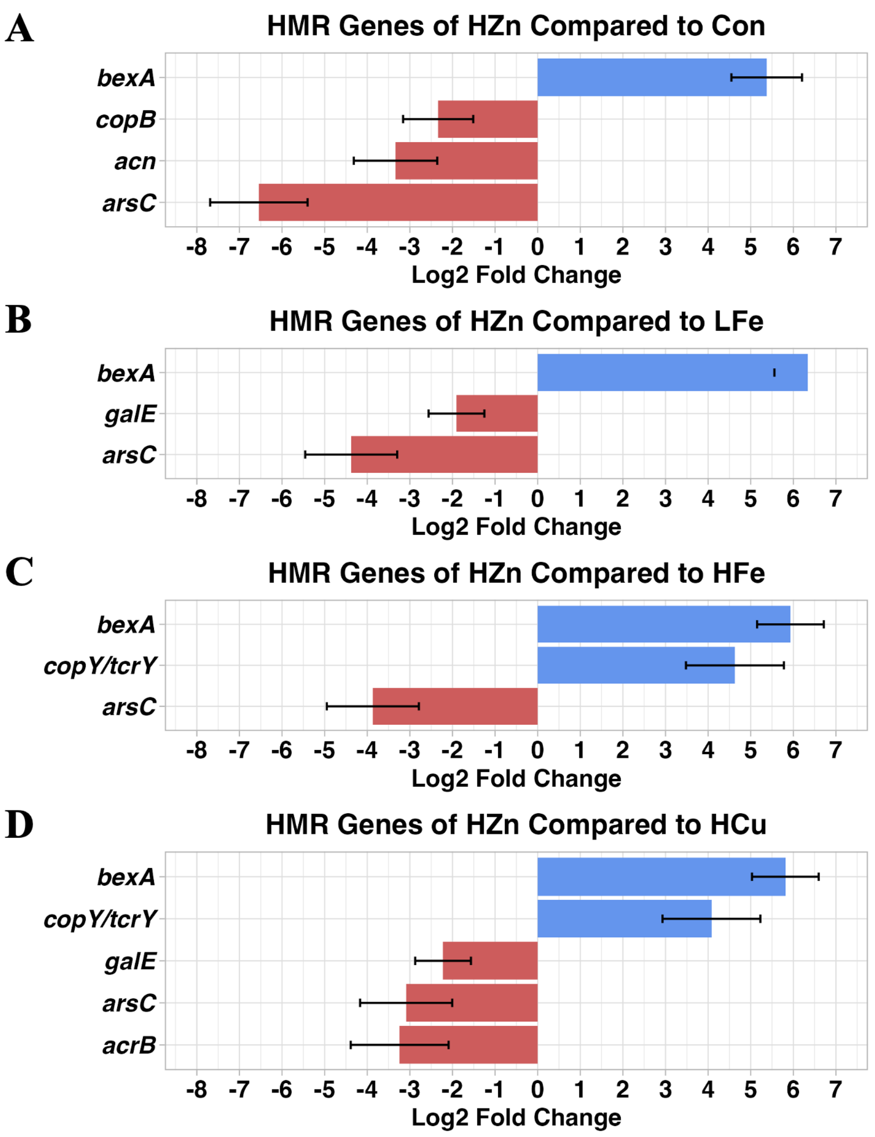

Figure S1. Differentially abundant heavy metal resistance (HMR) genes in the HZn group relative to Con (A), LFe (B), HFe (C), and HCu (D). Count data were analyzed using DESeq2. Genes with Benjamini–Hochberg adjusted $P < 0.05$ are shown. Differential abundance is presented as log2 fold change.

Dietary Iron and Metal Growth Promoters Differentially Modulate the Gut Resistome and E. coli Virulome in Weaned Pigs
Supplemental Figures

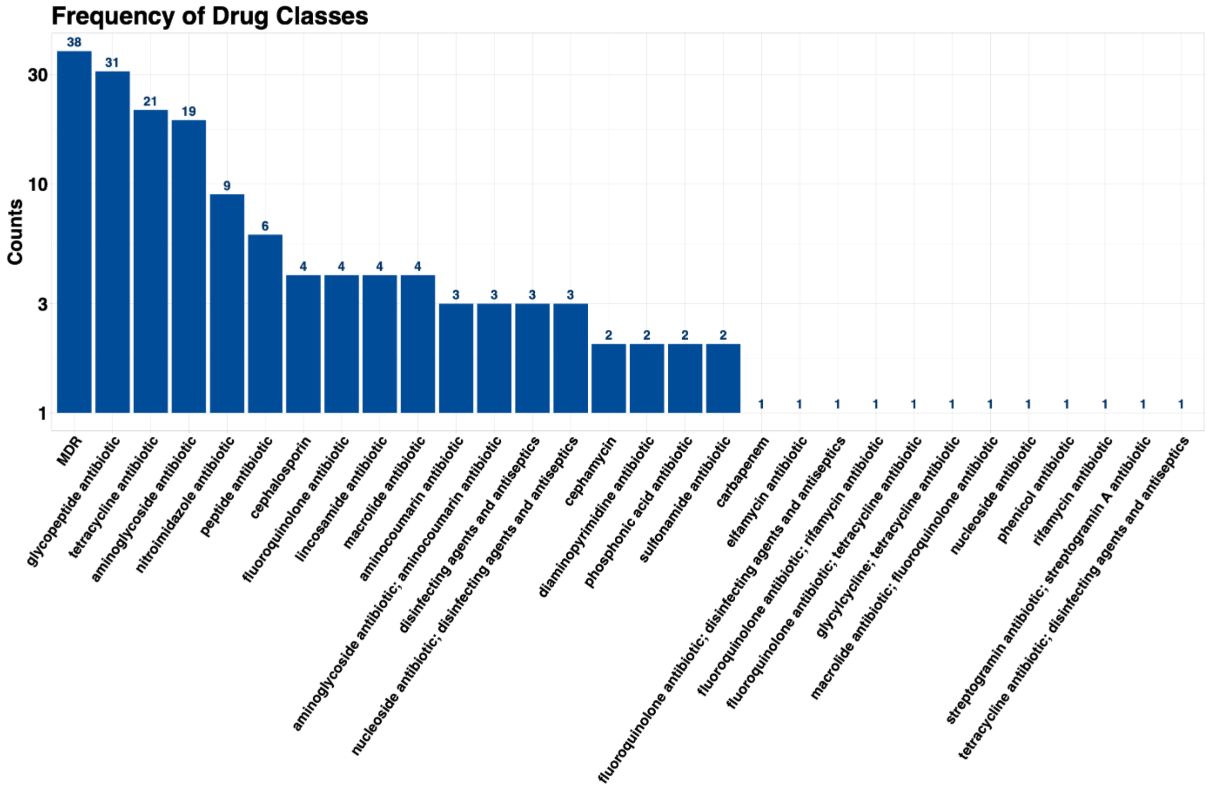

Figure S2. Distribution of antimicrobial drug classes in the fecal resistome across all metagenomes. Bars represent the number of unique antimicrobial resistance (AMR) genes detected within each drug class. MDR, multidrug resistance.

Dietary Iron and Metal Growth Promoters Differentially Modulate the Gut Resistome and E. coli Virulome in Weaned Pigs
Supplemental Figures

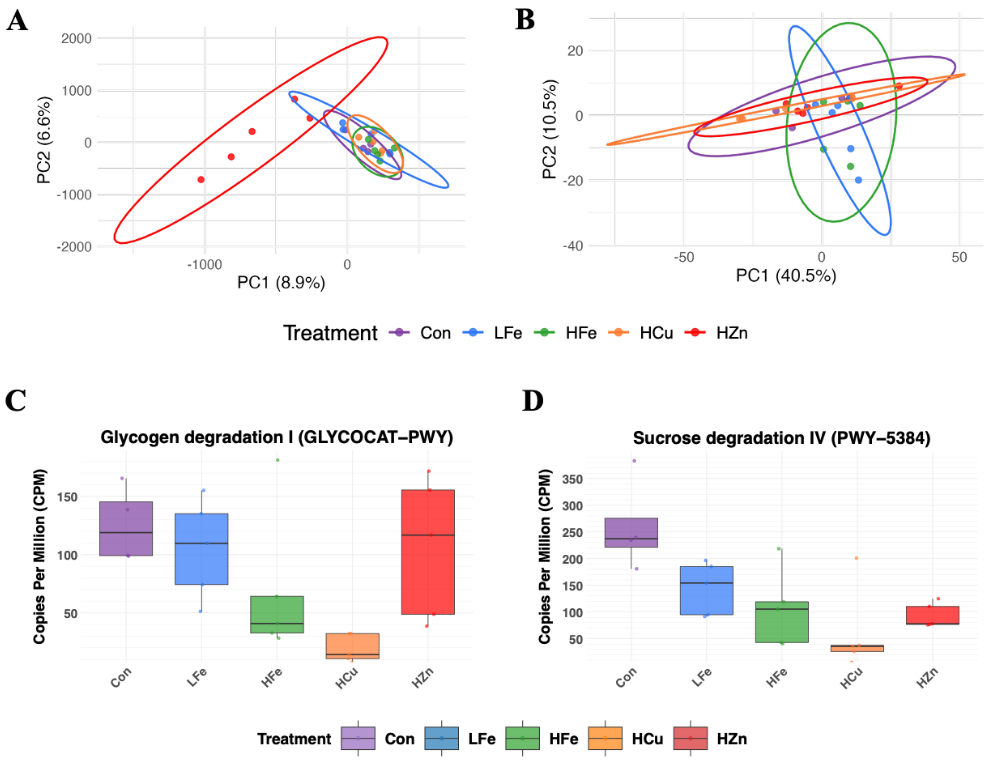

Figure S3. Principal component analysis (PCA) of centered log-ratio–transformed relative abundances of gene families (A) and functional pathways (B). Plots display the first two principal components with 95% confidence ellipses for each treatment group. Pathways significantly altered in the HCu diet relative to Con include glycogen degradation I (GLYCOCAT-PWY, C) and Sucrose Degradation IV (PWY-5384, D). Con, control diet; LFe, low-iron diet; HFe, high-iron diet; HCu, high-copper diet; HZn, high-zinc diet.

Dietary Iron and Metal Growth Promoters Differentially Modulate the Gut Resistome and E. coli Virulome in Weaned Pigs
Supplemental Figures

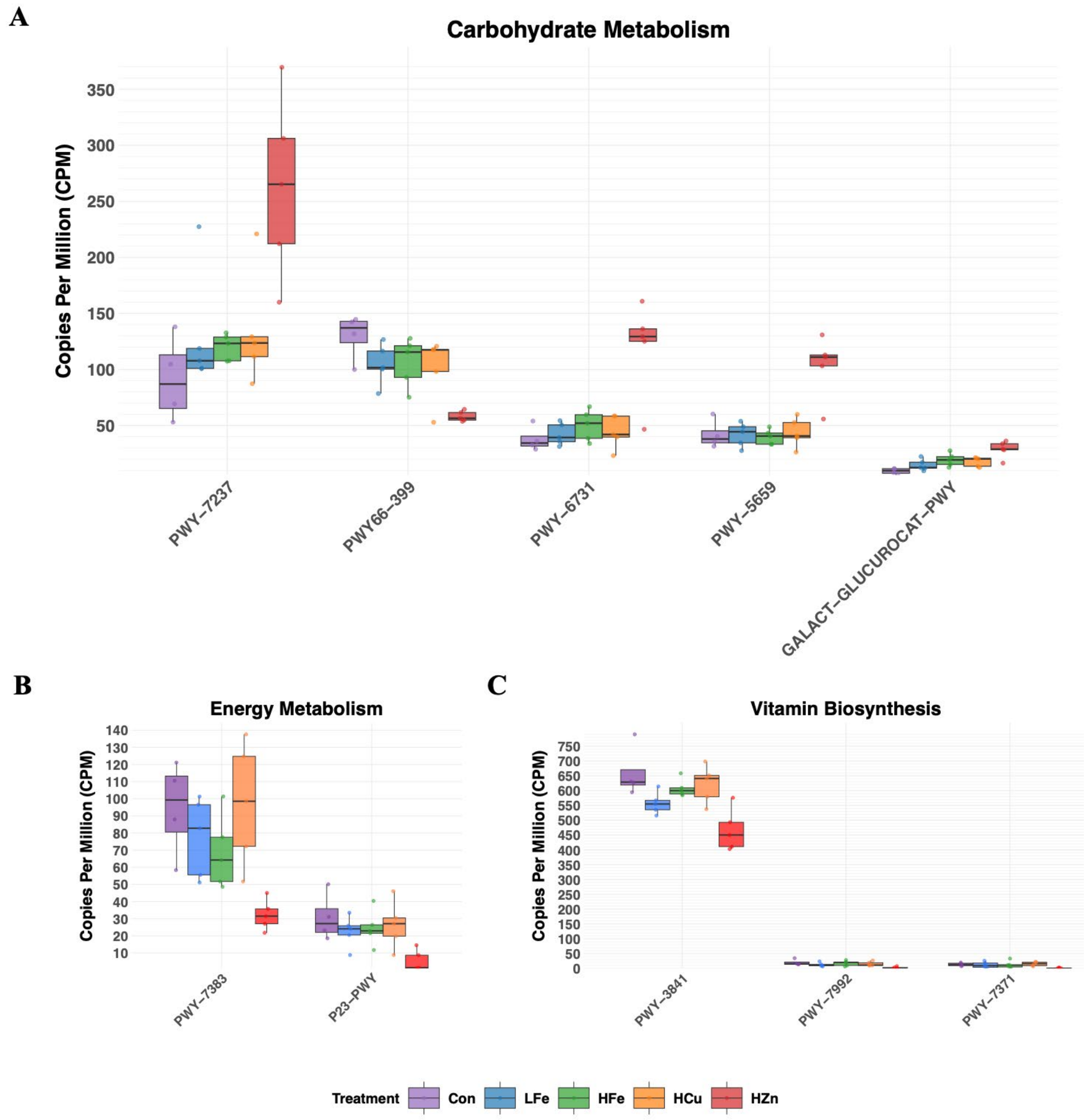

Figure S4. Functional pathways significantly altered by the HZn diet relative to Con. (A) Carbohydrate metabolism pathways: PWY-7237 (Myo-, chiro-, and scyllo-inositol degradation), PWY66-399 (Gluconeogenesis III), PWY-6731 (Starch degradation III), PWY-5659 (GDP-mannose biosynthesis), and GALACT-GLUCUROCAT-PWY (Superpathway of hexuronide and hexuronate degradation). (B) Energy metabolism pathways: PWY-7383 (Anaerobic energy metabolism) and P23-PWY (Reductive TCA cycle I). (C) Vitamin metabolism pathways: PWY-3841 (Folate transformations II), PWY-7992 (Superpathway of menaquinol-8 biosynthesis III), and PWY-7371 (1,4-dihydroxy-6-naphthoate biosynthesis II). Con, control diet; LFe, low-iron diet; HFe, high-iron diet; HCu, high-copper diet; HZn, high-zinc diet.

Dietary Iron and Metal Growth Promoters Differentially Modulate the Gut Resistome and E. coli Virulome in Weaned Pigs
Supplemental Figures

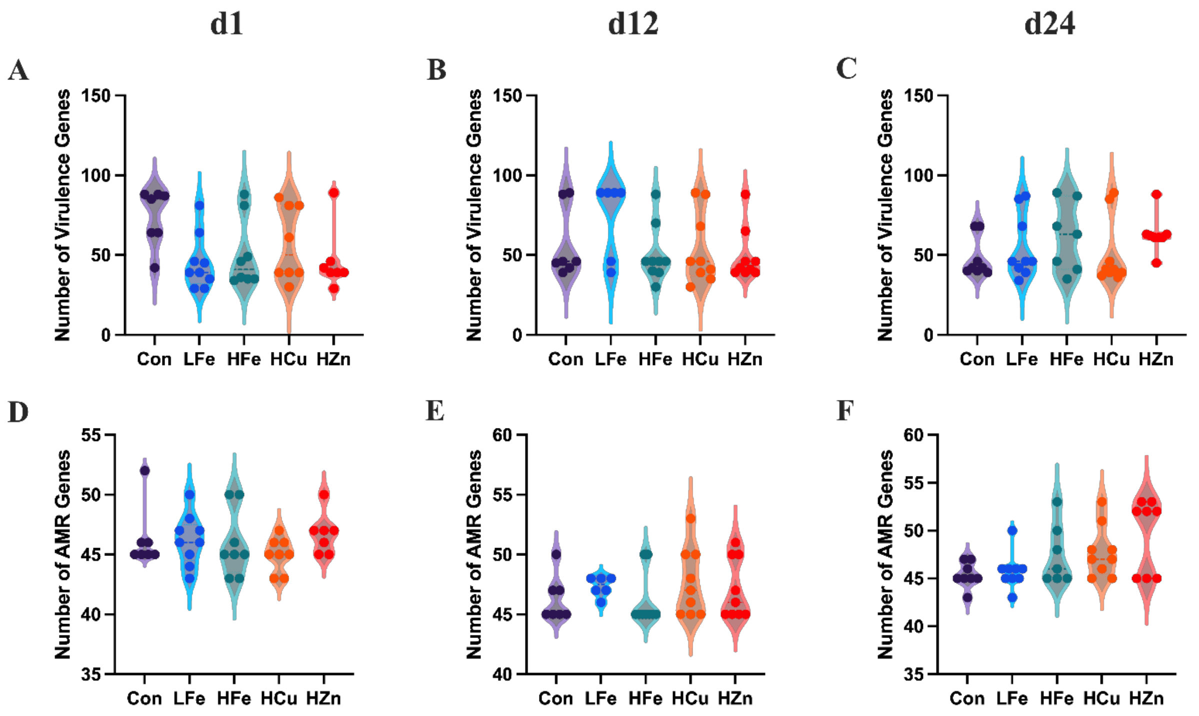

Figure S5. Number of virulence genes (A–C) and AMR genes (D–F) detected in isolated *E. coli* genomes on d 1, 12, and 24. Con, Control diet; LFe, Low Iron Diet; HFe, High Iron diet; HCu, High Copper diet; HZn, High Zinc diet
